# A highly nonlinear substitution-box (S-box) design using action of modular group on a projective line over a finite field

**DOI:** 10.1371/journal.pone.0241890

**Published:** 2020-11-12

**Authors:** Nasir Siddiqui, Fahim Yousaf, Fiza Murtaza, Muhammad Ehatisham-ul-Haq, M. Usman Ashraf, Ahmed M. Alghamdi, Ahmed S. Alfakeeh

**Affiliations:** 1 Department of Basic Sciences, University of Engineering and Technology (UET), Taxila, Punjab, Pakistan; 2 Sino-Pak Center for Artificial Intelligence, Pak-Austria Fachhochschule: Institute of Applied Sciences and Technology, Haripur, Khyber Pakthunkhwa (KPK), Pakistan; 3 Department of Computer Engineering, University of Engineering and Technology (UET), Taxila, Punjab, Pakistan; 4 Department of Computer Science, University of Management and Technology (UMT), Lahore (Sialkot), Punjab, Pakistan; 5 Department of Software Engineering, College of Computer Science and Engineering, University of Jeddah, Jeddah, Saudi Arabia; 6 Department of Information Systems, Faculty of Computing and Information Technology, King Abdulaziz University, Jeddah, Saudi Arabia; Wuhan University, CHINA

## Abstract

Cryptography is commonly used to secure communication and data transmission over insecure networks through the use of cryptosystems. A cryptosystem is a set of cryptographic algorithms offering security facilities for maintaining more cover-ups. A substitution-box (S-box) is the lone component in a cryptosystem that gives rise to a nonlinear mapping between inputs and outputs, thus providing confusion in data. An S-box that possesses high nonlinearity and low linear and differential probability is considered cryptographically secure. In this study, a new technique is presented to construct cryptographically strong 8×8 S-boxes by applying an adjacency matrix on the Galois field *GF*(2^8^). The adjacency matrix is obtained corresponding to the coset diagram for the action of modular group PSL(2,Z) on a projective line *PL*(*F*_7_) over a finite field *F*_7_. The strength of the proposed S-boxes is examined by common S-box tests, which validate their cryptographic strength. Moreover, we use the majority logic criterion to establish an image encryption application for the proposed S-boxes. The encryption results reveal the robustness and effectiveness of the proposed S-box design in image encryption applications.

## 1. Introduction

The significance of information security is expanding with time and the areas of communication and data transformation are becoming more and more complicated. It has now become very imperative to secure the transformation of essential data across insecure networks. The cryptographic algorithms provide the security and protection of the critical data and information getting transferred over insecure channels [1]. A block cipher is one of the most critical components of cryptography. Shannon [2] introduced the notion of modern cryptography in 1949. The block ciphers such as Data Encryption Standard (DES) [3] and Advanced Encryption Standard (AES) [4] rely on Shannon’s principle of confusion and diffusion [1, 5], where AES is the standard encryption technique approved by the National Institute of Standards and Technology (NIST). Confusion refers to the practice of making the correlation between the ciphertext and the key as intricate and complex as possible so that no one can understand the key by knowing the ciphertext. On the other hand, diffusion is the process of dissipating the influence of one plaintext bit on multiple ciphertext bits to obscure the statistical redundancies of the plaintext. Substitution-box (S-box) is the only dynamic component of the block ciphers that provides confusion through nonlinear mapping of inputs and outputs to adhere to the drill of encryption. The strength of an encryption method depends on the strength of the S-box [6, 7], which is evaluated using the NIST criteria. The nonlinearity of an S-box causes uncertainty in the output, which offers resistance against linear and differential cryptanalysis attacks [8]. An S-box design that yields high nonlinearity and low linear and differential probability is critical for a cryptosystem.

Many researchers laid their potential to design secure and reliable S-boxes. Rijndael proposed an algebraic S-box, called AES S-box [4], which is an integral part of the AES algorithm. Cui et al. [9] designed an improved AES S-box called Affine-Power-Affine (APA). In APA, the algebraic expression complexity of the AES S-box is increased from 9 to 253 terms while keeping the complexity of inverse S-box the same, i.e., 255. In [10], the authors presented Gray S-box for AES, which is generated by the addition of binary Gray code transformation to the standard AES S-box. The authors in [11] presented an improved AES S-box in which they enhanced the complexity of AES S-box algebraic expression with terms increasing from 9 to 255 and algebraic degree invariable. The improved AES S-box is capable of resisting against differential cryptanalysis with high dependable security. The authors in [12] modified the affine transformation of the AES S-box to minimize the time complexity of AES. In [13], the authors proposed a new S-box based on fractional linear transformation on the Galois field GF(2^8^), which can create confusion in the data. A few research studies [14–16] also put emphasis on using Cellular Automata (CA) for designing dynamic S-boxes and attained comparable cryptographic properties. In [17], the authors proposed two different S-boxes for AES by modifying the affine transformation matrices. In [18, 19], the authors proposed a variable and dynamic S-box mapping for AES using different irreducible polynomials. Thus, a different S-box is generated in each round of AES, which enhances the security of AES but only generates a limited number of S-boxes. The research works in [20–25] proposed key-dependent S-boxes based on secret keys for enhancing the security of AES.

Algebraic properties like group and rings [13, 26, 27], cubic fractional transformation [28], and Elliptic curve [29] have also been utilized to strengthen the differential probability of an S-box. In [30], Gaussian distribution and linear fractional transformation are used to design an S-box. The authors applied the Box-Muller transform, polarization decision, and central limit algorithm for generating the S-box. In [31], the authors proposed a method that generates highly non-linear n×n S-boxes (where 3 ≤ n ≤ 7). The authors used heuristic optimization to obtain the best S-box. Hussain et al. [32] developed a new construction method of S_8_ S-boxes by using the action of the symmetric group S_8_ on AES S-box that gives 40320 new S-boxes with the same strength as AES S-box. In [33], the authors proposed a hybrid scheme based on chaotic map and affine transformation to generate multiple S-boxes using rotational matrices. The research work in [34] utilized a chaotic map along with algebraic groups to proposed another hybrid method for generating non-linear S-boxes. A. Razzaq et al. [35] presented a new scheme for S-box design, which is based on the coset graphs and symmetric groups. Their proposed S-boxes attain strong cryptographic properties. In [36], the authors proposed an S-box design method based on single expression algebra to reduce the computational complexity of S-box design. Many researchers have utilized S-boxes in image encryption applications as well [37–41]. In [42], the authors proposed a novel method for image encryption in the Fresnelet domain. The proposed algorithm is dependent on the Fresnelet transform-based image decomposition along with an algebraic S-box. In [43], Shah et al. endorsed a standard norm to evaluate the fundamental types of S-boxes and analyze their competency for image encryption applications. Xiangjun et al. [44] presented a novel technique for color image encryption, which is based on coupled-map lattices (CML) and a fractional-order chaotic system.

In this study, we proposed a novel and efficient technique for designing 8×8 S-boxes based on the action of modular group PSL(2,Z) on a projective line *PL*(*F*_7_) over a finite field F_7_. For this purpose, we draw a coset diagram for the action of PSL(2,Z) on *PL*(*F*_7_) and form its adjacency matrix [45]. Then, we apply the adjacency matrix on Galois field *GF*(2^8^) elements using a set of different transformations to obtain bijective S-boxes. We inspect the cryptographic strength of the proposed S-boxes based on the NIST criteria using algebraic analyses such as nonlinearity, strict avalanche criterion, bit independent criterion, differential approximation probability, and linear approximation probability. Moreover, we utilized the proposed S-boxes for image encryption and perform statistical analyses on plain and encrypted images based on majority logic criterion [43, 46].

The rest of the paper organization is as follows. Section 2 explains the proposed method for S-box construction. Section 3 provides a discussion and comparison of the cryptographic strength of the proposed S-boxes. Section 4 discusses the application of the proposed S-boxes in image encryption along with its results. Finally, Section 5 concludes the findings of this research paper.

## 2. Proposed method for S-box design

**Fig 1:**
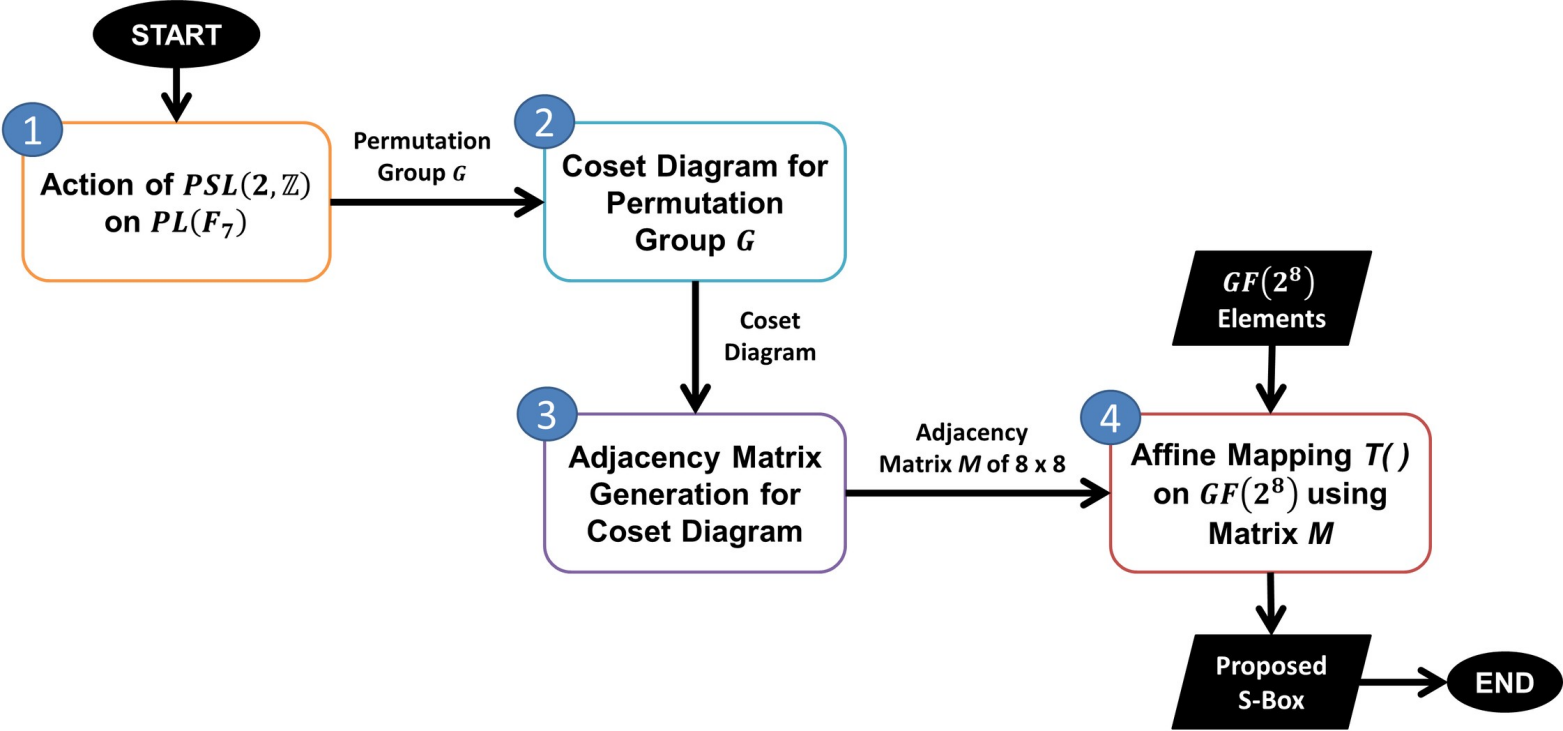
Proposed methodology for S-box design.

The proposed method for S-box construction is shown in Fig 1, which consists of four key steps. First, we perform an action of the modular group or projective special linear group PSL(2,Z) on a projective line *PL*(*F*_7_) over a finite field *F*_7_ to yield a permutation group *G*. After that, we draw a coset diagram for the permutation group *G* obtained corresponding to the action of PSL(2,Z) on *PL*(*F*_7_). Then, we generate an adjacency matrix corresponding to the obtained coset diagram. Finally, we use this adjacency matrix and apply an affine transformation on the Galois field elements followed by the addition of an 8-bit number to generate the final S-box.

The following sections briefly explain the key steps involved in the proposed S-box construction methodology.

### 2.1. Action of modular group PSL(2,Z) on projective line *PL*(*F*_7_)

The modular group PSL(2,Z) is a group comprises of all linear transformations $\to \frac{az+b}{cz+d}$, where a,b,c, and d are some integers satisfying the relation ad−bc=1.PSL(2,Z) is generated by linear fractional transformations $x:z\to \frac{-1}{z}$ and $y:z\to \frac{z-1}{z}$, which satisfy the relations x^2^ = y^3^ = 1. Eq (1) gives the finite representation of the modular group PSL(2,Z). A projective line over a Galois field F_n_ adds an extra point ∞ to F_n_ and is represented by PL(F_n_). Hence, a projective line PL(F_7_) over a field F_7_ contains eight points, which give rise to a coset diagram having eight vertices. Eq (2) defines a projective line PL(F_7_) over a finite field F_7_.

$$PSL(2,Z)=<x,y:x^{2}=y^{3}=1>$$ (1)

$$PL(F_{7})=F_{7}\cup \infty =\{0,1,2,3,4,5,6,\infty \}$$ (2)

The action of PSL(2,Z) on PL(F_7_) yields a permutation group G, which is generated by $\bar{x}$ and $\bar{y}$ given below.

$$\bar{x}=(0\;\infty )\;(1\;6)\;(2\;3)\;(4\;5)$$

$$\bar{y}=(0\;\infty \;1)\;(2\;4\;6)\;(3)\;(5)$$

### 2.2. Generation of coset diagram for permutation group G

**Fig 2:**
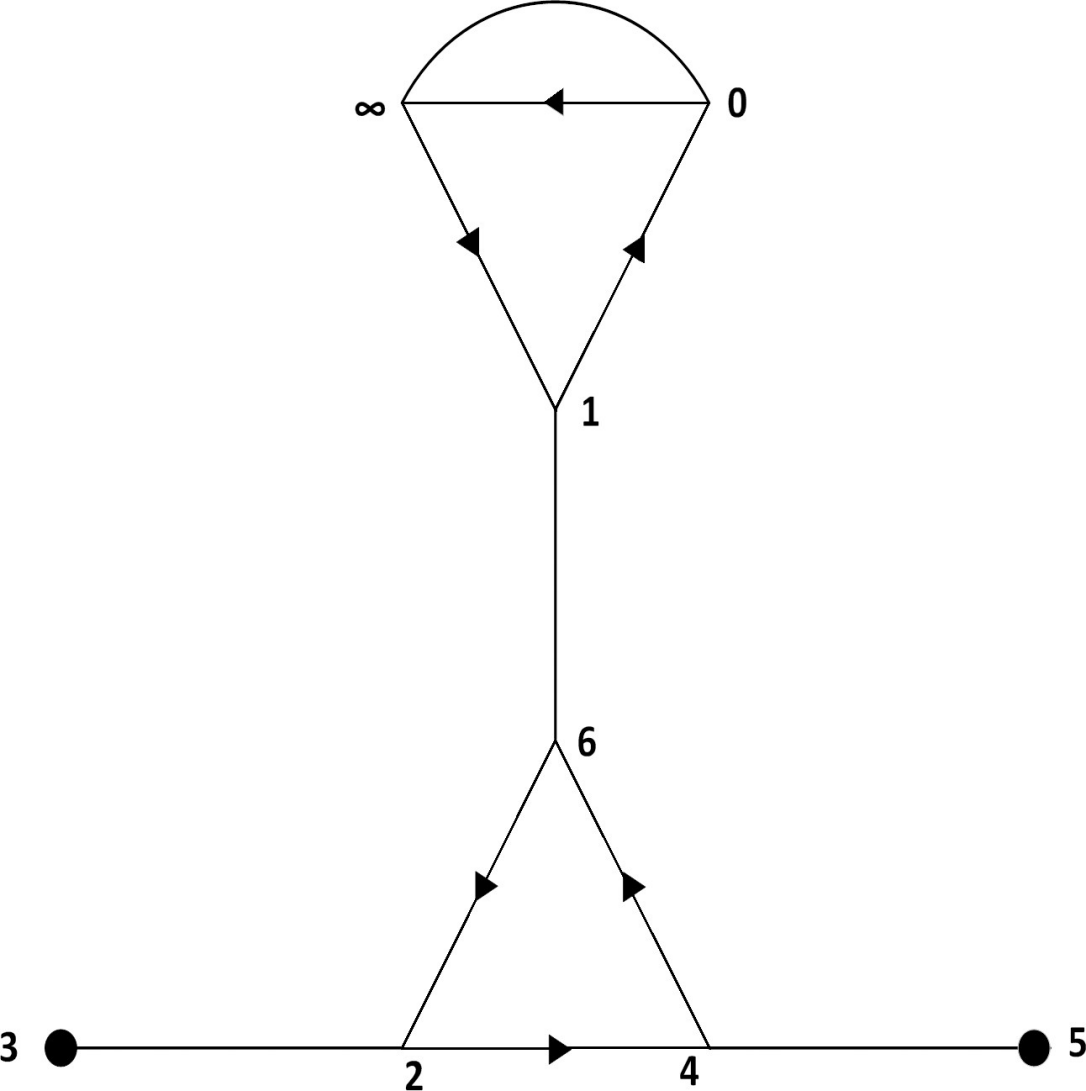
Coset diagram corresponding to the permutation group G.

After generating the permutation group G, we first draw a coset diagram using permutations $\bar{x}$ and $\bar{y}$. A coset diagram is a graphical way of representing the permutation action of a finitely-generated group [45]. Fig 2 shows the coset diagram obtained for the permutation group G. Since, $\bar{x}$ and $\bar{y}$ are of order 2 and 3 respectively, therefore, the generator $\bar{x}$ is denoted by an edge and the generator $\bar{y}$ is represented by a triangle. The vertices of the triangle are permuted counterclockwise and fixed points of $\bar{y}$ are denoted by heavy dots in the coset diagram.

### 2.3. Adjacency matrix generation for coset diagram

Next, we generate an adjacency matrix M from the coset diagram for the action of the PSL(2,Z) on PL(F_7_). The adjacency matrix for a directed graph G = (V,E), where V is the set of vertices and E is the set of edges, has a value 1 in its (i,j)th position if there exists an edge from v_i_ to v_j_, where v_1_,v_2_,…,v_n_ is an arbitrary listing of the vertices of the directed graph [47]. If we consider M = [m_ij_] as the adjacency matrix for the directed graph, then m_ij_ is defined as given below in Eq (3).

$$m_{ij}=\left\{ \begin{array}{l} 1,\;if\;(v_{i},v_{j})\;is\;an\;edge\;of\;G\\ 0,\;otherwise \end{array}\right.$$ (3)

In the coset diagram shown in Fig 2, the vertices are labeled as 0,1,2,3,4,5,6, and ∞. It can be seen from the figure that there exists an edge from 0 to ∞, therefore in the adjacency matrix, the entry of the 1st row and 8^th^ column is taken as 1 and all the remaining entries are set equal to zero in the 1st row. Similarly, in the 2^nd^ row of the adjacency matrix, the 1^st^ and 7^th^ elements are 1 because there exists an edge from 1 to 0 and 1 to 6. All other entries are equal to zero in this row. In the same way, by filling up the remaining entries in the matrix, we form an adjacency matrix M as given below.

$$M=\left[\begin{array}{c} 0\;0\;0\;0\;0\;0\;0\;1\\ 1\;0\;0\;0\;0\;0\;1\;0\\ 0\;0\;0\;1\;1\;0\;0\;0\\ 0\;0\;1\;1\;0\;0\;0\;0\\ 0\;0\;0\;0\;0\;1\;1\;0\\ 0\;0\;0\;0\;1\;1\;0\;0\\ 0\;1\;1\;0\;0\;0\;0\;0\\ 1\;1\;0\;0\;0\;0\;0\;0 \end{array}\right]$$

This adjacency matrix M is used in the later stage to generate the proposed S-boxes.

### 2.4. Affine mapping of Galois field using adjacency matrix

By applying the obtained adjacency matrix M on Galois field GF(2^8^), the obtained results are not distinctive. As the S-box elements must be unique, hence to attain the unique outcomes, we apply a transformation *T* on *GF*(2^8^). In this aspect, we propose a set of transformations T_k_, which is applied on the Galois field GF(2^8^) elements to generate multiple S-boxes, as shown in Eq (4).

$$T_{k}(t_{n})=Mt_{n}+\sum_{r\in I_{k}}t_{n+r(mod\;256)}$$ (4)

where, t_n_ represent the element of the Galois field GF(2^8^) in 8-bit binary form with n = 0,1,2,…,255, T_k_ represents a set of eight different transformations with *k* = 1,2,…,8, and *I*_*k*_ represents eight different sets of integer(s) given as: I_1_ = {1,2,3,…,128}, I_2_ = {2,4,6,…,128}, I_3_ = {4,8,12,…,128}, I_4_ = {8,16,24,…,128}, I_5_ = {16,32,48,…,128}, I_6_ = {32,64,96,128}, I_7_ = {64,128}, and I_8_ = {128}. For *k* = 8, we get *T*_8_(*t*_*n*_) = *Mt*_*n*_+*t*_*n*+128_(*mod* 256) from Eq (4). Similarly, taking *k* = 7 provides us *T*_7_(*t*_*n*_) = *Mt*_*n*_+*t*_*n*+64(*mod*256)_+*t*_*n*+128_(*mod* 256).

Table 1 shows the process of generating the S-box elements using transformation *T*_8_ (i.e., for *k* = 8). Likewise, other transformations (i.e., *T*_1_ to *T*_7_) can be applied on the Galois field *GF*(2^8^) elements to generate more S-boxes. *T*_7_(*t*_*n*_) = *Mt*_*n*_+*t*_*n*+64(*mod*256)_+*t*_*n*+128_(*mod* 256). Tables 2–9 present the proposed S-boxes generated as a result of applying transformations *T*_1_ to *T*_8_ on the Galois field *GF*(2^8^) elements, respectively, using the proposed scheme.

**Table 1:** Generation of the proposed S-box elements based on a transformation T_8_.

*t*_*n*_*ϵGF*(2^8^)	*T*_8_(*t*_*n*_) = *Mt*_*n*_+*t*_*n*+128(*mod* 256)_	S-box output
*t*_0_ = (0)_*d*_ = [0 0 0 0 0 0 0 0]^*t*^	***T***_**8**_(*t*_0_) = *Mt*_0_+*t*_0+128(***mod* 256**)_	[1 1 0 1 1 0 0 0]^*t*^ = (27)_*d*_
*t*_1_ = (1)_*d*_ = [1 0 0 0 0 0 0 0]^*t*^	***T***_**8**_(*t*_1_) = *Mt*_1_+*t*_1+128(***mod* 256**)_	[0 0 1 0 1 1 1 1]^*t*^ = (244)_*d*_
*t*_2_ = (2)_*d*_ = [0 1 0 0 0 0 0 0]^*t*^	***T***_**8**_(*t*_2_) = *Mt*_2_+*t*_2+128(***mod* 256**)_	[0 0 0 1 1 1 1 1]^*t*^ = (248)_*d*_
.	.	.
.	.	.
.	.	.
*t*_254_ = (254)_*d*_ = [0 1 1 1 1 1 1 1]^*t*^	***T***_**8**_(*t*_254_) = *Mt*_254_+*t*_254+128(***mod* 256**)_	[1 1 1 1 1 1 1 1]^*t*^ = (255)_*d*_
*t*_255_ = (255)_*d*_ = [1 1 1 1 1 1 1 1]^*t*^	***T***_**8**_(*t*_255_) = *Mt*_255_+*t*_255+128(***mod* 256**)_	[0 1 1 1 1 1 1 1]^*t*^ = (254)_*d*_

The subscript **d** represents a number in decimal form, whereas the superscript **t** donates the transpose of a vector.

**Table 2:** Proposed S-box (S1) in 16 × 16 matrix form—Generated with *T*_1_.

	*0*	*1*	*2*	*3*	*4*	*5*	*6*	*7*	*8*	*9*	*A*	*B*	*C*	*D*	*E*	*F*
***0***	0	244	165	89	22	147	2	105	71	186	163	184	48	30	39	49
***1***	164	94	211	143	219	187	7	100	72	46	181	231	132	252	174	154
***2***	216	88	200	113	151	65	199	9	224	102	215	67	19	99	189	220
***3***	96	138	226	177	82	179	13	59	108	81	230	63	32	50	190	8
***4***	104	35	24	79	75	131	153	145	37	26	155	92	85	222	16	120
***5***	23	196	52	5	31	208	250	180	202	87	28	198	51	139	109	106
***6***	248	95	27	29	93	170	133	58	166	78	124	176	221	157	210	90
***7***	218	251	235	175	207	34	66	117	17	209	135	107	21	12	127	253
***8***	84	161	213	54	125	168	150	61	204	228	4	146	247	98	152	173
***9***	242	191	121	233	212	232	169	80	229	238	217	40	255	126	225	171
***A***	223	68	140	56	10	25	42	160	188	15	97	129	115	118	128	144
***B***	194	70	77	185	116	243	74	112	130	159	91	36	33	178	110	38
***C***	246	41	114	192	162	172	14	55	214	249	62	76	148	11	193	203
***D***	241	134	86	44	60	206	141	239	240	20	237	57	183	156	236	47
***E***	53	18	1	142	245	83	103	69	158	101	167	122	45	73	136	43
***F***	201	3	195	64	123	197	119	6	234	227	137	111	182	149	205	254

**Table 3:** Proposed S-box (S2) in 16 × 16 matrix form—Generated with *T*_2_.

	*0*	*1*	*2*	*3*	*4*	*5*	*6*	*7*	*8*	*9*	*A*	*B*	*C*	*D*	*E*	*F*
***0***	27	142	18	248	210	43	73	221	83	93	133	69	101	166	124	122
***1***	52	44	134	23	109	47	156	51	206	31	250	239	20	202	28	57
***2***	135	111	227	17	66	6	197	207	149	21	127	254	3	218	235	64
***3***	155	76	249	37	153	55	172	75	11	85	16	203	41	104	24	192
***4***	68	216	200	56	118	19	189	144	199	160	25	151	215	129	15	224
***5***	191	164	211	233	126	132	174	171	7	80	232	219	181	40	238	72
***6***	159	108	230	36	243	82	13	112	190	38	178	32	226	185	70	96
***7***	228	71	163	146	168	22	2	61	39	173	98	48	165	54	161	0
***8***	84	244	89	213	247	30	49	152	105	150	125	147	184	4	204	186
***9***	194	138	177	77	33	50	8	110	59	74	116	179	63	91	130	81
***A***	229	46	231	217	212	187	100	169	154	225	255	252	143	121	242	94
***B***	188	102	67	97	10	65	9	42	220	128	115	99	113	140	223	88
***C***	79	114	246	35	120	193	148	222	162	131	145	14	214	26	92	62
***D***	175	195	201	251	253	205	182	12	123	34	117	119	234	209	107	137
***E***	198	237	240	87	180	141	60	208	183	139	106	236	241	196	5	86
***F***	176	167	158	78	58	103	245	170	45	157	90	136	53	95	29	1

**Table 4:** Proposed S-box (S3) in 16 × 16 matrix form—Generated with T_3_.

	*0*	*1*	*2*	*3*	*4*	*5*	*6*	*7*	*8*	*9*	*A*	*B*	*C*	*D*	*E*	*F*
***0***	0	29	165	95	133	147	93	105	71	176	163	78	210	30	221	49
***1***	164	175	211	251	66	187	207	100	72	107	181	209	127	252	21	154
***2***	216	79	200	35	153	65	75	9	224	92	215	26	16	99	85	220
***3***	96	5	226	196	250	179	31	59	108	198	230	87	109	50	51	8
***4***	104	113	24	88	199	131	151	145	37	67	155	102	189	222	19	120
***5***	23	177	52	138	13	208	82	180	202	63	28	81	190	139	32	106
***6***	248	89	27	244	2	170	22	58	166	184	124	186	39	157	48	90
***7***	218	143	235	94	7	34	219	117	17	231	135	46	174	12	132	253
***8***	84	142	213	18	103	168	245	61	204	122	4	101	136	98	45	173
***9***	242	64	121	3	119	232	123	80	229	111	217	227	205	126	182	171
***A***	223	192	140	41	14	25	162	160	188	76	97	249	193	118	148	144
***B***	194	44	77	134	141	243	60	112	130	57	91	20	236	178	183	38
***C***	246	56	114	68	42	172	10	55	214	129	62	15	128	11	115	203
***D***	241	185	86	70	74	206	116	239	240	36	237	159	110	156	33	47
***E***	53	54	1	161	150	83	125	69	158	146	167	228	152	73	247	43
***F***	201	233	195	191	169	197	212	6	234	40	137	238	225	149	255	254

**Table 5:** Proposed S-box (S4) in 16 × 16 matrix form—Generated with T_4_.

	*0*	*1*	*2*	*3*	*4*	*5*	*6*	*7*	*8*	*9*	*A*	*B*	*C*	*D*	*E*	*F*
***0***	0	29	165	95	22	58	2	170	124	186	166	184	210	30	221	49
***1***	164	175	211	251	219	117	7	34	135	46	17	231	127	252	21	154
***2***	216	79	200	35	151	145	199	131	155	102	37	67	16	99	85	220
***3***	96	5	226	196	82	180	13	208	28	81	202	63	109	50	51	8
***4***	104	113	24	88	75	9	153	65	215	26	224	92	189	222	19	120
***5***	23	177	52	138	31	59	250	179	230	87	108	198	190	139	32	106
***6***	248	89	27	244	93	105	133	147	163	78	71	176	39	157	48	90
***7***	218	143	235	94	207	100	66	187	181	209	72	107	174	12	132	253
***8***	84	142	213	18	125	69	150	83	167	228	158	146	136	98	45	173
***9***	242	64	121	3	212	6	169	197	137	238	234	40	205	126	182	171
***A***	223	192	140	41	10	55	42	172	62	15	214	129	193	118	148	144
***B***	194	44	77	134	116	239	74	206	237	159	240	36	236	178	183	38
***C***	246	56	114	68	162	160	14	25	97	249	188	76	128	11	115	203
***D***	241	185	86	70	60	112	141	243	91	20	130	57	110	156	33	47
***E***	53	54	1	161	245	61	103	168	4	101	204	122	152	73	247	43
***F***	201	233	195	191	123	80	119	232	217	227	229	111	225	149	255	254

**Table 6:** Proposed S-box (S5) in 16 × 16 matrix form—Generated with T_5_.

	*0*	*1*	*2*	*3*	*4*	*5*	*6*	*7*	*8*	*9*	*A*	*B*	*C*	*D*	*E*	*F*
***0***	0	29	165	95	22	58	2	170	71	176	163	78	48	90	39	157
***1***	235	94	218	143	66	187	207	100	135	46	17	231	127	252	21	154
***2***	216	79	200	35	151	145	199	131	224	92	215	26	19	120	189	222
***3***	52	138	23	177	250	179	31	59	28	81	202	63	109	50	51	8
***4***	104	113	24	88	75	9	153	65	37	67	155	102	85	220	16	99
***5***	226	196	96	5	13	208	82	180	230	87	108	198	190	139	32	106
***6***	248	89	27	244	93	105	133	147	166	184	124	186	221	49	210	30
***7***	211	251	164	175	7	34	219	117	181	209	72	107	174	12	132	253
***8***	84	142	213	18	125	69	150	83	204	122	4	101	247	43	152	73
***9***	195	191	201	233	119	232	123	80	137	238	234	40	205	126	182	171
***A***	223	192	140	41	10	55	42	172	188	76	97	249	115	203	128	11
***B***	86	70	241	185	141	243	60	112	237	159	240	36	236	178	183	38
***C***	246	56	114	68	162	160	14	25	214	129	62	15	148	144	193	118
***D***	77	134	194	44	74	206	116	239	91	20	130	57	110	156	33	47
***E***	53	54	1	161	245	61	103	168	158	146	167	228	45	173	136	98
***F***	121	3	242	64	169	197	212	6	217	227	229	111	225	149	255	254

**Table 7:** Proposed S-box (S6) in 16 × 16 matrix form—Generated with T_6_.

	*0*	*1*	*2*	*3*	*4*	*5*	*6*	*7*	*8*	*9*	*A*	*B*	*C*	*D*	*E*	*F*
***0***	27	142	54	165	210	43	173	39	83	93	22	168	101	166	71	228
***1***	52	44	185	226	109	47	38	190	206	31	82	243	20	202	108	159
***2***	72	238	227	17	219	232	197	207	171	174	127	254	233	211	235	64
***3***	224	15	249	37	151	25	172	75	144	189	16	203	56	200	24	192
***4***	68	216	104	41	118	19	85	11	199	160	55	153	215	129	76	155
***5***	191	164	218	3	126	132	21	149	7	80	6	66	181	40	111	135
***6***	57	28	230	36	239	250	13	112	51	156	178	32	23	134	70	96
***7***	122	124	163	146	69	133	2	61	221	73	98	48	248	18	161	0
***8***	84	244	95	53	247	30	157	45	105	150	103	58	184	4	167	176
***9***	194	138	196	241	33	50	139	183	59	74	141	180	63	91	237	198
***A***	137	107	231	217	119	117	100	169	12	182	255	252	251	201	242	94
***B***	62	92	67	97	14	145	9	42	222	148	115	99	35	246	223	88
***C***	79	114	140	113	120	193	128	220	162	131	65	10	214	26	102	188
***D***	175	195	121	143	253	205	225	154	123	34	187	212	234	209	46	229
***E***	81	130	240	87	179	116	60	208	110	8	106	236	77	177	5	86
***F***	186	204	158	78	147	125	245	170	152	49	90	136	213	89	29	1

**Table 8:** Proposed S-box (S7) in 16 × 16 matrix form—Generated with T_7_.

	*0*	*1*	*2*	*3*	*4*	*5*	*6*	*7*	*8*	*9*	*A*	*B*	*C*	*D*	*E*	*F*
***0***	18	248	0	161	73	221	48	98	133	69	61	2	124	122	146	163
***1***	134	23	96	70	156	51	32	178	250	239	112	13	28	57	36	230
***2***	227	17	72	238	197	207	219	232	127	254	171	174	235	64	233	211
***3***	249	37	224	15	172	75	151	25	16	203	144	189	24	192	56	200
***4***	104	41	68	216	85	11	118	19	55	153	199	160	76	155	215	129
***5***	218	3	191	164	21	149	126	132	6	66	7	80	111	135	181	40
***6***	202	20	159	108	31	206	243	82	47	109	190	38	44	52	226	185
***7***	166	101	228	71	93	83	168	22	43	210	39	173	142	27	165	54
***8***	89	213	1	29	49	152	136	90	125	147	170	245	204	186	78	158
***9***	177	77	86	5	8	110	236	106	116	179	208	60	130	81	87	240
***A***	231	217	137	107	100	169	119	117	255	252	12	182	242	94	251	201
***B***	67	97	62	92	9	42	14	145	115	99	222	148	223	88	35	246
***C***	140	113	79	114	128	220	120	193	65	10	162	131	102	188	214	26
***D***	121	143	175	195	225	154	253	205	187	212	123	34	46	229	234	209
***E***	91	63	198	237	74	59	180	141	50	33	183	139	138	194	241	196
***F***	4	184	176	167	150	105	58	103	30	247	45	157	244	84	53	95

**Table 9:** Proposed S-box (S8) in 16 × 16 matrix form—generated with T_8_.

	*0*	*1*	*2*	*3*	*4*	*5*	*6*	*7*	*8*	*9*	*A*	*B*	*C*	*D*	*E*	*F*
***0***	27	244	248	89	133	147	93	105	124	186	166	184	210	30	221	49
***1***	235	94	218	143	66	187	207	100	135	46	17	231	127	252	21	154
***2***	24	88	104	113	153	65	75	9	155	102	37	67	16	99	85	220
***3***	52	138	23	177	250	179	31	59	28	81	202	63	109	50	51	8
***4***	200	35	216	79	199	131	151	145	215	26	224	92	189	222	19	120
***5***	226	196	96	5	13	208	82	180	230	87	108	198	190	139	32	106
***6***	165	95	0	29	2	170	22	58	163	78	71	176	39	157	48	90
***7***	211	251	164	175	7	34	219	117	181	209	72	107	174	12	132	253
***8***	1	161	53	54	103	168	245	61	167	228	158	146	136	98	45	173
***9***	195	191	201	233	119	232	123	80	137	238	234	40	205	126	182	171
***A***	114	68	246	56	14	25	162	160	62	15	214	129	193	118	148	144
***B***	86	70	241	185	141	243	60	112	237	159	240	36	236	178	183	38
***C***	140	41	223	192	42	172	10	55	97	249	188	76	128	11	115	203
***D***	77	134	194	44	74	206	116	239	91	20	130	57	110	156	33	47
***E***	213	18	84	142	150	83	125	69	4	101	204	122	152	73	247	43
***F***	121	3	242	64	169	197	212	6	217	227	229	111	225	149	255	254

## 3. Performance analysis of proposed S-boxes

In this section, we validate the cryptographic strength of the proposed S-boxes (presented as S1-S8 in Tables 2–9, respectively) by commonly used parameters, which include: nonlinearity [48], bit independence criterion (BIC) [13, 49], strict avalanche criterion (SAC) [49], linear and differential approximation probabilities [50]. The nonlinearity of an n-variable Boolean function represents the minimum distance of the reference function from the set of all n-variable affine functions. Mathematically, the relationship between the nonlinearity of an n-variable Boolean function and the Walsh Hadamard transform of that function is defined as $N(f)=2^{n-1}-2^{\frac{n}{2}-1}$ [48]. For GF(2^8^), the optimal value of nonlinearity is 120. The BIC quantifies the independence between the avalanche variables. To test this criterion, the variables are compared pairwise to extract knowledge about the independence of these variables. The input bits are complemented individually, and the output vectors are analyzed for independence. The SAC depends upon the variation of the input outcomes and output bits. An S-box satisfies the SAC only if changing a single input bit yields a change in half of the output bits. An ideal S-box has the SAC value equal to one-half, i.e., 0.5 [49]. The linear approximation probability (LAP) identifies the probability of bias for a given S-box, whereas the differential approximation probability (DAP) measures the differential uniformity of an S-box [50]. The mathematical description of the LAP and DAP are given in Eqs (5) and (6) respectively.

$$LAP=\underset{\Gamma_{x},\Gamma_{y}}{\max}|\frac{\#\{x|x.\Gamma_{x}=S(x).\Gamma_{y}\}}{2^{n}}-\frac{1}{2}|$$ (5)

where, Γ_x_ and Γ_y_ are input and output masks respectively, x is the set of all probable input values and is the number of S-box elements.

$$DAP(\Delta x\to \Delta y)=\left[\frac{\#\{x\in X|S(x)\pm S(x\pm \Delta x=\Delta y\}}{2^{n}}\right]$$ (6)

where, Δx represents input differential, Δy is output differential, X is the set of all probable inputs, and 2^n^ is the number of its elements.

As the research on S-box construction is becoming increasingly vital, numerous researchers have designed tools for testing S-box performance [51–53], which are based on the NIST criteria. These tools provide ease to the researchers in testing and verifying the S-box parameters. For validating the proposed S-boxes in our paper, we utilized the S-box testing tool presented by the authors in [51]. Table 10 summarizes the numerical values of the performance metrics obtained for the proposed S-boxes and compares these results with those obtained for some well-known S-boxes.

**Table 10:** Numerical results of the S-box testing parameters obtained for our proposals in comparison with the existing S-boxes.

S-box		Nonlinearity	SAC	BIC	LAP	DAP
**Proposed**	**S1**	112.0	0.4951	112.0	0.0625	0.0156
	**S2**	112.0	0.4951	112.0	0.0625	0.0156
	**S3**	112.0	0.4970	112.0	0.0625	0.0156
	**S4**	112.0	0.5000	112.0	0.0625	0.0156
	**S5**	112.0	0.4995	112.0	0.0625	0.0156
	**S6**	112.0	0.4953	112.0	0.0625	0.0156
	**S7**	112.0	0.4960	112.0	0.0625	0.0156
	**S8**	112.0	0.4953	112.0	0.0625	0.0156
AES [4]		112.0	0.5058	112.0	0.0625	0.0156
APA [9]		112.0	0.4987	112.0	0.0625	0.0156
Gray [10]		112.0	0.505	111.46	0.0664	0.0156
Zahid et al. [28]		107.0	0.497	103.5	0.1560	0.0390
Farwa et al. [41]		103.5	0.5065	103.3	0.1328	0.0468
Aboytes et al. [16]		112.0	0.4998	112.0	0.0625	0.0156
Khan et al. [30]		111.0	0.5036	110.0	0.0781	0.0234
Hayat et al. [29]		100.0	0.5007	104.1	0.0390	0.1250

By investigating the results presented in Table 10, it can be stated that the proposed S-boxes achieve high nonlinearity and BIC value (i.e., 112), which is the maximum possible value achieved with any of the existing S-boxes till now. The proposed S-boxes also satisfy the SAC test by achieving a near-optimal value of 0.5. Furthermore, the maximum value of linear and differential approximation probability for all the proposed S-boxes is 0.0625 and 0.0156 respectively, which is better than or comparable to those obtained for existing S-boxes as shown in Table 10. Overall, the proposed S-boxes yield a high nonlinearity and low linear and differential probability values, thus offering strong resistance against linear and differential cryptanalysis. As a result, we conclude that the proposed S-boxes possess strong algebraic and cryptographic properties, thus capable of demonstrating effective performance in different security applications.

The proposed S-box design provides an additional advantage over the standard AES S-box in the sense that it can generate multiple S-boxes using the single adjacency matrix. If the affine matrix is changed adhering to the defined criteria in our proposed method, a new set of S-boxes can be generated with the same cryptographic strength as AES S-box. In this aspect, the additional cost of generating the affine matrix is trivial, thus the overall complexity per S-box generation tends to be insignificant. As a result, it can be stated that the proposed S-box design method is computationally feasible and the obtained S-boxes are cryptographically strong as the AES S-box.

## 4. Application of proposed S-box in image encryption

As an application of the proposed S-boxes, we perform image encryption using proposed S-boxes and assess their strength and robustness in image encryption based on the majority logic criterion (MLC) [43, 46]. We take a standard 8-bit Baboon image of size 512 × 512 as a plain gray-level image and encrypt this image independently using AES S-box and the proposed S-boxes (i.e., S2, S5, and S7). For this purpose, we substitute every pixel value in the image with the corresponding value in the S-box, which scrambles the visual information in the image and provides image encryption. We perform one round encryption on plain Lena image and carry out some statistical analyses on plain and encrypted images. These analyses include entropy, energy, correlation, contrast, and homogeneity analysis [46]. Table 11 provides a brief description of these statistical parameters, which are computed using a gray-level co-occurrence matrix (GLCM) [46]. The numerical results of these parameters are provided in Table 12.

**Table 11:** Statistical analysis parameters with description and formulae.

Statistical Analysisy	Description	Formulae
Entropy	Measures the randomness in an image and provides information about the image texture/gray level	$H=-\sum_{k=0}^{n}p(r_{k})\log_{2}p(r_{k})$
Energy	Quantifies the energy in an image by using GLCM	$E=\sum_{a}\sum_{b}p^{2}(a,b)$
Correlation	Evaluates the independence between the plain and encrypted images	$K=\sum_{a,b}\frac{\left(a-\mu a)(b-\mu b)p(a,b\right)}{\sigma_{a}\sigma_{b}}$
Contrast	Determines the diffusion on an image and identifies the objects in an image	$C=\sum_{a}\sum_{b}{\left(a-b\right)}^{2}p(a,b)$
Homogeneity	Determines the characteristics of the distribution exhibited by the elements in the GLCM with respect to the GLCM diagonal	$H=\sum_{a}\sum_{b}\frac{p(a,b)}{1-|a-b|}$

* *p*(*a*,*b*) is the number of GLCMs.

**Table 12:** Comparison of statistical analysis parameters obtained for plain and encrypted Baboon images.

Statistical Analysis	Plain Image	Encrypted Baboon Image with different S-boxes				
		AES	S2	S5	S7	S8
**Entropy**	7.358	7.358	7.358	7.358	7.358	7.358
**Energy**	0.089	0.016	0.016	0.016	0.016	0.016
**Correlation**	0.830	0.014	0.018	0.011	0.006	0.026
**Contrast**	0.617	10.50	9.808	10.05	9.863	9.849
**Homogeneity**	0.787	0.400	0.406	0.401	0.407	0.402

It can be observed from Table 12 that the proposed S-box (i.e., S2, S5, S7, and S8) provides effective image encryption results and the obtained parameters are mostly comparable to the AES S-box. The entropy value obtained for the encrypted images using the proposed S-boxes is 7.358, which is near to the ideal value of 8. As the entropy measures the randomness in an image, hence, the nonlinear substitution of input and output elements in the image amplifies its randomness. The energy measure value of the plain Baboon image is 0.089. After encrypting this plain image with the proposed S-boxes, we achieve an energy value of 0.016, which is comparable to the AES S-box energy value. The smaller energy measure indicates the efficient performance of the proposed S-boxes in image encryption. To show the linear independence between the plain and encrypted images, we find out the correlation coefficient between both images. A coefficient value near 0 represents no or weak linear correlation between both images. In the case of image encryption with the proposed S-boxes, the correlation between the plain image and its encrypted form is 0.018, 0.011, 0.006, and 0.026 using S2, S5, S7, and S8 S-boxes, respectively, as shown in Table 12. These statistics represent that there is a weak linear correlation among the input and output pixel values. Hence, the proposed S-boxes provide good encryption properties such as confusion and diffusion. Moreover, the proposed S-boxes achieve a high contrast value (more than 9.8). A constant image has a contrast value of 0. Generally, a high value of contrast means more randomness in the image. Due to the nonlinearity of mapping, the objects in the image are distorted entirely after applying the S-box. That is why the high value of contrast in the encrypted image shows strong encryption. Finally, we perform the homogeneity analysis to measure the closeness of the distributed elements of GLCM to its diagonal. Table 12 also displays the results of this statistical analysis, where the proposed S-boxes achieve an acceptable homogeneity value, which results in the favor of having better encryption. So, overall, the image encryption results obtained for the proposed S-boxes are comparable to the state-of-the-art results as shown in Table 12.

**Fig 3:**
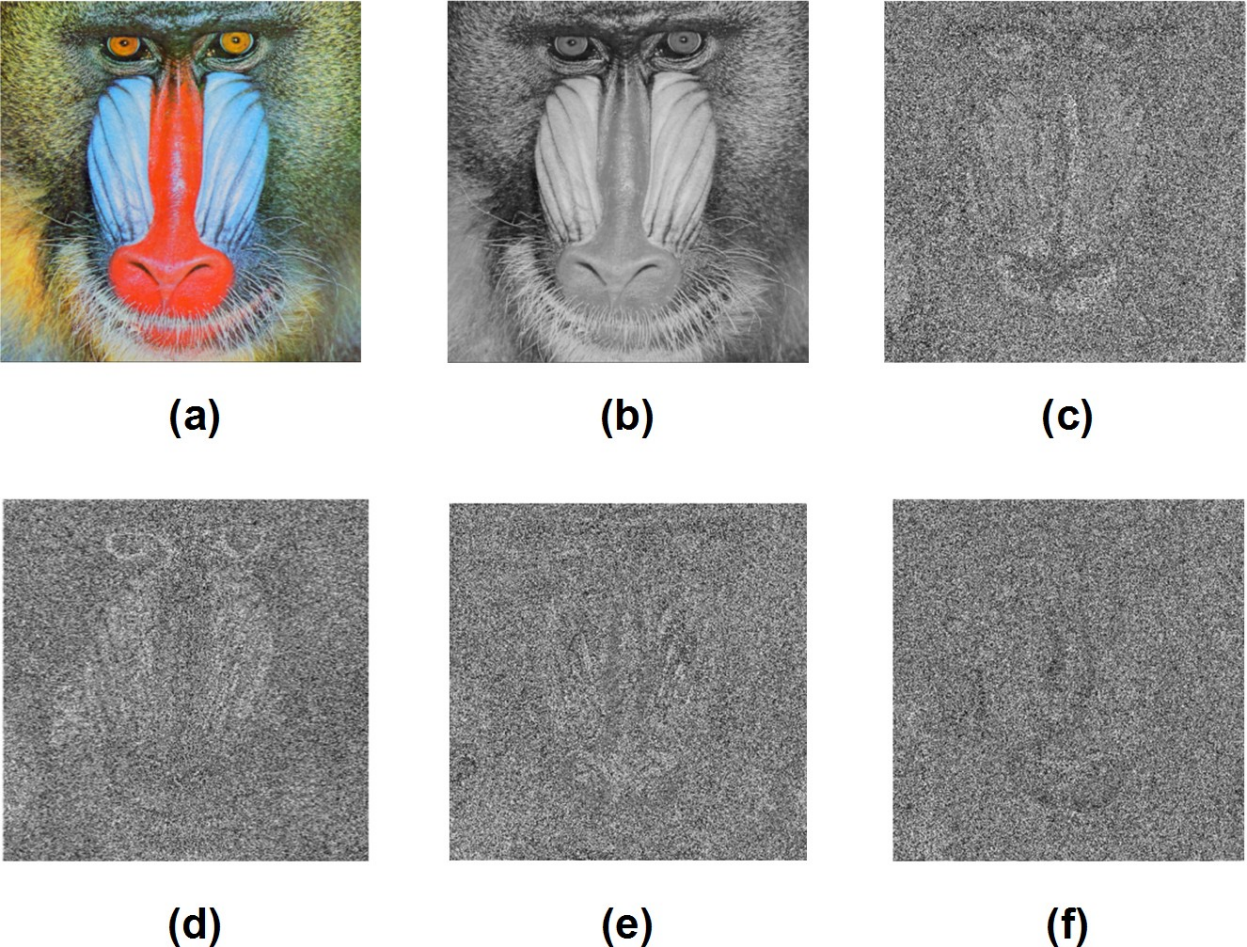
Image encryption using the proposed S-box design. (a) Plain Baboon color image, (b) Plain Baboon gray-scale image, (c) Encrypted Baboon image using AES S-box, (d) Encrypted Baboon image using proposed S-box (S2), (e) Encrypted Baboon image using proposed S-box (S5), and (f) Encrypted Baboon image using proposed S-box (S7).

Fig 3 provides a visual demonstration of encrypted images using different S-boxes. It can be observed from the figure that the proposed S-boxes effectively hide the visual information contained in the plain image, which indicates their excellent performance in image encryption. Therefore, we conclude that the proposed S-box design can be successfully utilized for image encryption applications.

## 5. Conclusions

In this paper, we present a novel matrix-based approach for the construction of highly nonlinear S-boxes. For this purpose, we first construct an adjacency matrix of size 8×8 corresponding to the coset diagram obtained for the action of projective special linear group PSL(2,Z) on a projective line PL(F_7_) over a finite field F_7_. Afterward, we apply this adjacency matrix on the Galois field GF(2^8^) using a set of algebraic transformations to generate the final 8×8 S-boxes. We analyze the algebraic strength of the proposed S-boxes with common S-box tests, which validate their cryptographic strength. Furthermore, we also utilize the proposed S-boxes for image encryption and use statistical analyses to investigate the performance of our proposed S-boxes, which demonstrate the effectiveness of the proposed S-box design in image encryption applications. In the future, the proposed scheme can be expanded to generate *n*×*n* S-boxes using different action groups and adjacecy matrices of size *n*×*n*.
